# Change in subjective well-being over 20 years at two Norwegian medical schools and factors linked to well-being today: a survey

**DOI:** 10.1186/s12909-019-1476-3

**Published:** 2019-02-04

**Authors:** Christian Sletta, Reidar Tyssen, Lise Tevik Løvseth

**Affiliations:** 1 Department of Mental Health, Faculty of Medicine and Health Sciences, Norwegian University of Science and Technology, Trondheim, Norway; 2 Department of Behavioural Sciences, Medicine Institute of Basic Medical Sciences Faculty of Medicine University of Oslo, Oslo, Norway; 3 Department of Research and Development, Division of Mental Health Care, St. Olavs Trondheim University Hospital, Postbox 3250 Torgarden, NO-7006 Trondheim, Norway

**Keywords:** Medical school, Medical student, Subjective well-being, Life satisfaction, Stress, Social support

## Abstract

**Background:**

There is a lack of studies on factors in the curriculum, study environment and individual differences that can promote well-being among medical students as a response to the frequent reports on the negative health effects of study demands among medical students worldwide.

**Objective:**

This study investigates differences in well-being among today’s Norwegian medical students compared with students 20 years ago, the most important predictors of well-being today, and whether there have been any changes in the levels of some of these factors since the period analysed.

**Methods:**

We analysed cross-sectional survey data among all medical students (63.9%, *N* = 1044/1635) at two medical faculties with different curriculums (traditional and integrated) in Norway in 2015 (STUDMED 2015). We used comparison data from a longitudinal survey among medical students from the same medical faculties in 1993 to 1999: the NORDOC project (T1 = 89%, T2 = 72% and T3 = 68%). Differences in subjective well-being and correlates by demographic, curriculum, and study environment factors among the present students were tested by *t*-tests and stepwise linear regression analysis.

**Results:**

Students today scored lower on their levels of subjective well-being than students 20 years ago. The difference was found among female and males in different study stages. The final model showed that subjective well-being today was associated with self-esteem (β = .98, *p* < .001) and social support from medical school friends (β = .22, *p* < .001), a partner (β = .08, *p* = .020) or other family members (β = .04, *p* = .041), as well as perception of medical curriculum and environment (β = −.38, *p* < .001), personal competence (β = −.40, *p* < .001), finance/accommodation (β = −.22, *p* < .001) and perceived exam stress (β = −.26, p < .001).

**Conclusions:**

The results show a decrease in subjective well-being among medical students and, in particular, among female students. The faculties should pay attention to the factors identified in the study environment and curriculum associated with subjective well-being in order to promote their student’s well-being and stimulate health and academic performance.

## Background

Most studies on well-being among medical students and junior doctors have been on negative aspects of study and work stress and its consequences, such as burnout, anxiety and depression [1, 2]. Though study stress among medical students can be potentially impairing, the study of medicine is also a process that stimulates learning, competence and growth. It is important to increase knowledge about factors that are linked to satisfaction and well-being, as they can affect the students’ health and academic performance [3–5]. The data from the last systematic investigation of subjective well-being among Norwegian medical students are more than 20 years old [6, 7]. Societal changes, as well as continuous curriculum revisions at the medical faculties in Norway, can lead to new influential factors on medical students’ well-being. We need studies that investigate changes in students’ well-being over time and its correlates.

Subjective well-being is defined as people’s overall evaluations of their lives and their emotional experiences [8]. The subjective well-being construct encompasses affective elements (moods and emotions) in terms of 1) positive affect and 2) negative affect; it also encompasses one cognitive element: 3) life satisfaction [9, 10], which is defined as the individual’s overall judgement of their own life assessed by one’s own standards, based on one’s own values and interests. In contrast to traditional models of mental health that have focused on maladaptive behaviour and factors that contributes to stress and ill-health [11], subjective well-being is a part of the emerging literature in mental health that focuses on adaptive behaviour and flourishing [12]. Even though subjective well-being and depressive symptomatology is closely and negatively correlated, the emphasise on psychological distress and negative affect contributes to a negative focus on medical student stress and ill-health among scholars [2, 13, 14]. Contrarily, focusing on well-being among students provides more comprehensive knowledge on both stress and well-being among medical students. Factors related to subjective well-being include differences in the medical curriculum, study environment and individual differences among the students with respect to perceived social support and personality.

With respect to factors related to the curriculum, a recent review of longitudinal integrated clerkships demonstrated that these types of clerkships provide better academic outcomes for students compared with traditional block rotations [15], and that that students in curricula with problem based learning (PBL) have more favourable perceptions of the learning environment [16]. Changing from several grading categories to a pass/fail system is associated with lower levels of stress and burnout [17], and this can increase satisfaction and decrease perceived stress among the students [18, 19]. In Norway, all faculties have a 6-year curriculum. Some faculties emphasize that medical students meet patients as early as their first semester and have PBL as a way of learning from year 1 (integrated curriculum), while others have less patient contact until year 3 (traditional curriculum). There are also differences in the number of exams and grading: the faculty in this study, with integrated curriculum, has one annual exam in the first four study years with a pass/fail assessment. By contrast, the faculty with ‘traditional’ curriculum have more frequent exams and grades. Concordant with other studies of well-being and even perceived skills [20–22], a Norwegian study from 2005 showed more positive attitudes among students after transitioning from a traditional to an integrated curriculum [23].

The study environment can also contribute to students’ subjective well-being. Several studies have shown that social activities [24], social relations [25], a network of social support [26] and a stable economy and relationship status [27, 28] are important for student well-being. A Norwegian longitudinal study showed that those medical students who reported sustained high levels of life satisfaction were students that experienced medical school as less interfering with their social and personal life [6]. Important social life factors include support from peers as well as a partner and family. As for individual differences, self-esteem is a strong predictor of overall life satisfaction among students worldwide [29, 30]. Self-esteem is defined as a person’s sense of self-worth, and as subjective well-being pertains to people’s overall evaluations of their lives, these outcomes are positively associated with one another [30]. A previous study among Norwegian medical students showed that the level of self-esteem was lower than in the general population and that male medical students scored even lower than female medical students did at that time [31]. Self-esteem is also important to prevent negative self-report bias in studies of subjective well-being, since low self-esteem resembles neuroticism [32] and negative affectivity [33].

There could have been changes in the level of subjective well-being over the last two decades due to demographic changes in the medical student population. The rate of female students has increased, from about 60% of first-year students in 1993 to 71.6% in 2015 in our two respective faculties [34]. National reports in Norway from 2015 and 2017 show gender differences regarding satisfaction with one’s own health, with men being more satisfied, as well as an increase in symptoms of anxiety and depression among young Norwegian women since 2010 [35, 36].

The last comprehensive study of medical students’ health and well-being in Norway was derived from NORDOC, The longitudinal study of Norwegian medical students and doctors from two decades ago [6, 7]. Within that time span, there has been minimal change in the curricula between traditional and integrated curriculum in the two Norwegian medical faculties we aim to study, but the context has changed both in terms of an increased amount of female medical students, as well as societal changes.

The aim of the current study is to investigate levels of subjective well-being among medical students at two Norwegian medical schools and to investigate the most important factors related to subjective well-being among medical students today (2015). Additionally, we want to compare the levels of student well-being today with that of medical students two decades ago (1993–1999) and identify any possible changes between the two that could explain the differences in well-being over the same period.

## Methods

### Setting

All registered medical students at the time of the data collection at two universities in Norway were considered eligible and invited to participate in a cross-sectional survey on the effects of study curriculum and study conditions on contentment and mental health among Norwegian medical students (STUDMED). The study was conducted in two Norwegian medical faculties, both of which run a curriculum lasting for 6 years with the same entry criteria, the same number of students and the same share of female students. However, contentment and training periods differed between the faculties. One faculty runs an integrated curriculum and employs a problem-based learning model involving early patient contact and the integration of the original pre-clinical and clinical subjects. The students have annual exams with a pass/fail grading system. The other faculty runs a traditional model with a slightly shorter initial pre-clinical phase in the first 2 years without patient contact; this is separated from the following four-year clinical phase. The students with a traditional curriculum have frequent exams graded from A to F.

The sample included 1635 students (*N* = 709 with integrated curriculum/FacInt, and *N* = 926 with traditional curriculum/FacTrad). In total, 34.7% (*N* = 568) were male, and 65.3% (*N* = 1067) were female students.

### Study design

The data collection took place in February and March 2015. The Regional Ethics Committee and the administration of each faculty approved the project. Prior to the survey, participants received information about the study in lectures and a project Facebook page. Invitations and log-on information to access the web-based questionnaire were sent by mail. The participants had to provide their written consent to participate before entering their responses anonymously into the web-based questionnaire. Reminders were provided once each week by e-mail to those who had not responded to the questionnaire. In addition, the project provided general reminders about the project in lectures and on the project page by union representatives for medical students at each faculty during the survey period. The project referred to a psychiatrist, the Students Health Service and an emergency service in case of any distress in individual students related to answering the questionnaire.

To enable the investigation of changes in subjective well-being, the current study includes longitudinal data from the NORDOC survey (*N* = 453), which includes a cohort of medical students from the same faculties (FacInt: *N* = 156, FacTrad: *N* = 297), at three points of time: at the study start in 1993 (T1), then at 1996 (T2) and 1999 (T3). The response rates in NORDOC were 89% at T1, 72% at T2, and 68% at T3 [6]. In our article, data from NORDOC is used as cross-sectional data. More information about the NORDOC study is fully described elsewhere [6].

### Measures

The questionnaire consisted of items concerning education, health, study environment and demographics. The present study was based on a selection of variables relevant to the current foci.

The dependent variable, *Subjective Well-being* (SWB) was measured by a validated instrument of four items on different dimensions of well-being [37]. This includes a cognitive element (life satisfaction), positive affect element (happy and strong) and negative affect element (unhappy and tired). Item 1) At present do you mostly feel strong and fit, or tired and worn out, and item 2) Would you say you are usually cheerful or dejected, were scored on a 7-point scale from 1 (very tired/dejected/not happy) to 7 (strong and fit/cheerful/happy). Item 3) Would you say you are mostly happy or dejected and item 4) Over the last 14 days, have you suffered from nervousness, was scored on a 5-point scale from 1 (not at all) to 5 (very much). Item 4 was reversed prior to construction of the index. As the number of response categories varied between the items, the score was transformed to 0–10 by the algorithm: X = (Y-1) × 10/(Z-1), where X was the new score, Y the original score and Z the number of response categories [38]. The index was based on the mean score of the four items. High mean score indicates high SWB (*N* = 4, α = .80). As only three of the four items were applied in the NORDOC project, the comparison between STUDMED and NORDOC was done using items 1, 2 and 4.

### Independent variables

#### Demographics and individual differences

*Gender* (1 = male, 2 = female)*. Faculty* (1 = FacInt, 2 = FacTrad). *Year of study* was categorized into 1st stage (1-4th semester), 2nd stage (5-8th semester) and 3rd stage (9th–12th semester). 1st stage was used as baseline, and dummy variables were created for the 2nd and 3rd stages.

*Age when entering medical school* was dichotomised with a cut-off at 21 years (1 = < 21 years, 2 = ≥ 21 years). In Norway, there are two quotas for admission to higher education, where age is one of the requirements for those who apply with only their high school diploma.

*Accommodation* consisted of two items: 1) living alone (0 = not alone, 1 = alone) and 2) living with friends (0 = not with friends, 1 = with friends).

*Parents’ educational level* was categorized into 1 (up to college/university) and 2 (college/university).

*Self-esteem* included seven items derived from a subscale of Basic Character Inventory (BCI) about vulnerability (or neuroticism) [32, 39]. They have previously been validated [40] and the items focus on both low general self-esteem and high general self-esteem, with answers ranging on a scale from 1 (disagree) to 4 (agree). High mean sum scores indicate high general self-esteem (*N* = 7, α = .86). Table 1 provides a description of the independent variables.

**Table 1:** Description of the independent variables

Variable	STUDMED		NORDOC	
	Range	Mean (SD) or percentages	Range	Mean (SD) or percentages
Gender (female)		70.9%		53.4%
Faculty (FacTrad)		50.2%		67.0%
2nd stage		33.0%		
3rd stage		33.1%		
Age at study start (≥ 21 years)		48.7%		
Education mother (higher education)		82.8%		
Education father (higher education)		82.2%		
Living alone		20.7%		
Living with friends		44.3%		
Self esteem	1–4	2.89 (.66)		
PMSS – (ColdThreat) Medical school is cold and threatening	1–5	2.46 (.71)		
PMSS – (WorkComp) Worries about work and competence	1–5	2.99 (.83)		
PMSS – (FinAcc) Worries about finance and accommodation	1–5	2.22 (1.06)		
Social support				
SSpar (parents)	1–5	4.23 (.96)		
SSmed (medical school friends)	1–5	3.86 (.95)		
SSofr (other friends)	1–5	3.64 (.95)		
SSfam (other family members)	1–5	3.59 (1.13)		
SSptn (partner)	1–5	4.53 (.76)		
SSadm (study administration)	1–5	2.25 (1.03)		
Perceived exam stress	1–5	3.37 (.93)		
Satisfaction with supervision	1–5	2.91 (.83)		
Failing a medical exam (Failed once or more)		9.6%		

#### Study model factors

*Satisfaction with supervision* included four items on perceived quality of clinical supervision, which ranged on a five-point scale from 1 (poor) to 5 (good).

*Failing medical school exam* was measured by a single item on whether the student ever had failed an exam at medical school (1 = yes/0 = no). Participants that had not taken an exam (*N* = 93) were coded as missing.

*Perceived Medical School Stress (PMSS)* [41] is an instrument used to monitor stress among medical students related to the medical school curriculum, personal competence, social/recreational life and finance/living situations. A modified version of 13 items meeting Norwegian standards was used [31]. Each item is scored on a five-point scale from 1 (strongly disagree) to 4 (strongly agree), where a high score indicates high stress. A factor analysis with Varimax rotation confirmed a three factor solution which explained 56.16% of the total variance [42, 43]. Each factor was entered the model as independent variables: 1) Medical school is cold and threatening (ColdThreat, 5 items, α = .82); 2) Worries about work and competence (WorkComp, 5 items, α = .72); and 3) Worries about finance and accommodation (FinAcc, 3 items α = .72),. For comparison between NORDOC and STUDMED, a mean sum score was calculated of the 8 items which were used in NORDOC T2, T3 and STUDMED.

*Perceived exam stress* consisted of three newly made items for the current project. Participants were asked to rate from 1 (very small degree) to 5 (very high degree) the following statements: 1) I have worries concerning exams beyond the examination period; 2) I perceive exams as stressful; and 3) I perceive exams as demanding. A high mean sum score indicates high Perceived exam stress (α = .83).

#### Social life factors

*Social support* [44, 45] was measured by the following questions: 1) ‘How much is each of the following willing to spend time to make studies easier for you?’; 2) ‘How much can you confide in and how much are the following persons willing to listen if you want to talk with them about your personal problems?’; and 3) ‘How much can you trust the following persons to help you when the study situation gets difficult?’. The questions was to be answered in relation to six categories of support (parents, medical school friends, other friends, other family members, partner and study administration) on a five point scale from 1 (never) to 5 (very often/always). Participants’ responses to the three support items were added across each peer category and included in the model as six independent variables of social support: 1) Parents (SSpar, α = .78); 2) medical school friends (SSmed, α = .84), 3); other friends (SSofr, α = .78), 4); other family members (SSfam, α = .85); 5) partner (SSptn, α = .98); and 6) study administration (SSadm, α = .79). Individuals answering ‘not relevant’ were coded as 0 to avoid many missing values in further analyses.

### Statistics

We analysed differences and changes in subjective well-being based on gender, faculty and year of study by calculating means or proportions with 95% confidence intervals (CIs) and by independent samples *t*-tests. Differences in continuous variables were analysed using *t*-tests and one-way analysis of variance (ANOVA). The differences in well-being were also analysed with effect sizes (Cohen’s *d*: 0.02–0.49 = small effect; 0.50–0.79 = moderate effect; ≥ 0.08 = large effect) [46]. In order to test the representativeness of faculty, gender and study stage of the sample we used the Chi-square test of independence (χ^2^). We conducted a stepwise multiple linear regression analysis with SWB as the dependent variable to investigate covariates of SWB according to faculty and ran preliminary analyses to ensure that there was no violation of the assumptions of normality, linearity and multicollinearity. To manage missing values, we applied pairwise deletion. We used stepwise regression analyses to investigate the relative influence by explained variance of demographics, individual factors, study curriculum, environment and self-esteem, respectively. Except for gender, faculty and year of study, which were included in all analyses, we used a cut-off of *p* ≤ .10 for variables to be entered in the next model to avoid type II errors [47, 48]. We used SPSS statistical software package version 23 to analyse the data (Table 2).

**Table 2:** Correlation between variables

	1	2	3	4	5	6	7	8	9	10	11	12	13	14	15	16	17	18	19	20	21	22	23
1 Subjective Well-Being																							
Demographic																							
2 Gender	−.11^a^																						
3 Faculty	−.07	.02																					
4 2nd stage	.00	−.09^a^	−.04																				
5 3rd stage	.01	.02	−.03	−.49^a^																			
Personal																							
6 Age at study start	−.03	−.04	−.05	−.02	−.01																		
7 Education mother	.01	.02	−.08^a^	.05	−.02	−.07																	
8 Education father	.05	−.03	−.01	.00	.04	−.09^a^	.36^a^																
9 Living alone	−.02	−.02	.08^a^	.02	−.02	−.05	−.05	−.02															
10 Living with friends	.04	−.02	−.10^a^	.02	−.14^a^	−.11^a^	.09^a^	.07	−.45^a^														
Study characteristics																							
11 Satisfaction with supervision new	.19^a^	−.09^a^	−.13^a^	.03	.01	.03	.05	−.00	.03	.02													
12 Failing a medical exam	−.14^a^	−.03	−.12^a^	−.02	.08	.13^a^	−.11^a^	−.03	.05	−.04	.04												
Study environment																							
Perceived Medical School Stress																							
13 Medical School is cold and threatening	−.37^a^	.00	.26^a^	−.07	.13^a^	.04	−.11^a^	−.07	.01	−.10^a^	−.21^a^	.14^a^											
14 Worries about work and competence	−.45^a^	.21^a^	−.06	.03	−.06	−.05	.05	.03	−.06	.02	−.23^a^	.05	.00										
15 Worries about finance and accomodation	−.22^a^	.06	.10^a^	−.02	−.05	.08	−.03	−.05	−.04	.03	−.02	.07	.00	.00									
Social support																							
16 Parents	.20^a^	.06	−.03	.03	−.04	−.03	.14^a^	.08	.02	.10^a^	.07	−.06	−.11^a^	−.07	−.16^a^								
17 Medical school friends	.36^a^	.08^a^	−.08^a^	.02	.01	−.09^a^	.15^a^	.08^a^	−.01	.15^a^	.13^a^	−.21^a^	−.35^a^	−.10^a^	−.13^a^	.36^a^							
18 Other friends	.25^a^	.13^a^	−.04	−.03	.03	.00	.08^a^	.03	.01	.13^a^	.13^a^	−.10^a^	−.18^a^	−.12^a^	−.11^a^	.41^a^	.49^a^						
19 Other family members	.22^a^	.08	.02	−.03	.03	−.06	.10^a^	.01	−.01	.07	.13^a^	−.13^a^	−.13^a^	−.08^a^	−.08	.47^a^	.36^a^	.51^a^					
20 Partner	.07	.05	−.03	.03	.13^a^	.13^a^	−.03	−.01	−.21^a^	−.30^a^	.04	−.01	.06	−.03	−.00	−.00	−.07	−.06	−.00				
21 Study administration	.12^a^	−.05	−.02	−.03	−.03	−.03	.02	−.03	−.05	.05	.12^a^	.04	−.08^a^	−.11^a^	−.05	.10^a^	.11^a^	.14^a^	.11^a^	.04			
22 Perceived exam stress	−.46^a^	.24^a^	−.23^a^	.01	−.02	.03	−.03	−.03	−.02	−.06	−.14^a^	.21^a^	.21^a^	.49^a^	.10^a^	−.07	−.17^a^	−.13^a^	−.12^a^	.01	−.12^a^		
23 Self esteem	.62^a^	−.15^a^	−.05	−.04	.06	.02	.00	,08^a^	.02	.02	.16^a^	−.05	−.24^a^	−.44^a^	−.18^a^	.15^a^	.28^a^	.23^a^	.16^a^	.12^a^	.11^a^	−.41^a^	

^a^Correlation is significant at the 0.01 level (2-tailed)

## Results

The response rates in STUDMED were 63.9% (1044/1635) in total; 73.4% (520/709) at FacInt and 56.6% (524/926) at FacTrad (χ^2^ = 10.66, df = 1, *p* = .001). The STUDMED sample consisted of 740 female and 304 male participants, aged from 19 to 46 years (mean = 24.9 years, SD = 3.11). A response analysis showed that there were more female (740/1067) than male respondents (304/568, χ^2^ = 9.17, df = 1, *p* = .003). There was no significant difference in response rates based on study stage. Compared with the mean age in NORDOC T1 of 21.8 years (SD = 2.86), the 1st year students in STUDMED had a mean age of 22.5 years (SD = 3.36, *p* = .040).

The analyses showed a lower SWB score in STUDMED compared with NORDOC at all three stages of medical school: 1st stage mean STUDMED = 6.35 (SD = 1.85) vs NORDOC T1 = 7.60 (1.35), *p* < .001; 2nd stage mean STUDMED = 6.33 (2.01) vs NORDOC T2 = 7.16 (1.59), *p* < .001; and 3rd stage mean STUDMED = 6.44 (1.97) vs NORDOC T3 = 7.09 (1.61), *p* < .001). Table 3 shows mean scores in SWB according to gender and study stage.

**Table 3:** Comparison of STUDMED and NORDOC on Subjective Well-Being based on study stage and gender

SWB	Female		Male	
Study stage	STUDMED *N* = 740	NORDOC *N* = 259	STUDMED *N* = 304	NORDOC *N* = 194
	Mean (SD) (95%CI)	Mean (SD) (95%CI)	Mean (SD) (95%CI)	Mean (SD) (95%CI)
T1	6.22 (1.88) (5.99–6.45)	7.70 (1.27)*** (7.44–7.96)	6.74 (1.73) (6.37–7.11)	7.49 (1.45)** (7.17–7.81)
T2	6.04 (2.02) (5.77–6.31)	7.24 (1.55)*** (6.92–7.56)	6.88 (1.89) (6.54–7.22)	7.17 (1.62) (6.73–7.61)
T3	6.29 (2.00) (6.04–6.54)	6.90 (1.54)* (6.55–7.25)	6.80 (1.87) (6.42–7.18)	7.33 (1.68) (6.90–7.76)

*= *p* value less than 0.05

**= *p* value less than 0.01

***= *p* value less than 0.001

95%*CI* Confidence Interval

The lower scores in SWB in STUDMED compared with NORDOC is most apparent among female medical students. The analyses showed that female students scored lower on SWB at all stages: 1st stage (*p* < .001, *d* = .92); 2nd stage (*p <* .001, *d* = .67); and 3rd stage (*p* = .014, *d* = .34) than NORDOC T1, T2 and T3. For male students, there was a difference in 1st stage only (*p* = .003, *d* = .47).

In the STUDMED sample 82.1% of the participants had a score > 5.00 (mean = 6.55, SD = 1.82). Students at FacInt scored higher on SWB (mean = 6.67, SD =1.80) than FacTrad (mean = 6.43, SD = 1.82, *p* = .034). In the total sample, male participants scored significantly higher on SWB (mean = 6.86, SD = 1.80) than females (mean = 6.42, SD = 1.81, *p* < .001)*.*

### Regression analysis

Stepwise linear regression was performed to investigate the relative influence of the independent variables’ on SWB. Table 4 presents the univariate associations. The significant adjusted effects in Model 1 were female gender and FacTrad (adjusted R^2^ = .012); in Model 2, they were female gender and FacTrad (adjusted R^2^ = .011). In Model 3, female gender, FacTrad, ‘satisfied with supervision’ and ‘failing medical school’ had a significant adjusted effect (adjusted R^2^ = .064). The factors of PMSS (ColdThreat, WorkComp, FinAcc), Social support by SSmed and SSptn, and Perceived exam stress in Model 4 showed adjusted R^2^ = .455. Introducing Self-esteem in Model 5 resulted in an adjusted R^2^ = .533 and significant association with ColdThreat, WorkComp, FinAcc, SSmed, SSptn, SSfam, Perceived exam stress and Self-esteem (Table 3). Post-hoc analysis without the students who had not taken any exams did not alter the results. The stepwise analysis showed that Model 4 increased most of the explained variance, with a change in adj. R^2^ = .391. Additional analysis showed that the effect of gender in was mediated by the effect of WorkComp and Perceived exam stress. The effect of faculty was mediated by the effects of ColdThreat, FinAcc and SSmed. There was interaction with gender and SSfam and no interaction with gender or faculty in any of the other significant factors variables included in the model.

**Table 4:** Linear regression analysis. Dependent variable: Subjective Well-Being

Variable	Unadjusted			Model 1			Model 2			Model 3			Model 4			Model 5		
	*B?*	*95% CI*	*p*	*b*	*95% CI*	*p*	*b*	*95% CI*	*p*	*b*	*95% CI*	*p*	*b*	*95% CI*	*p*	*b*	*95% CI*	*p*
Demographic																		
Gender (1 = male, 2 = female)	**−.43**	−.67- -.19	.000	**−.43**	−.67- -.19	.000	**−.43**	−.67- -.18	.001	**−.38**	−.63- -.13	.003	−.02	−.22–.18	.843	.06	−.12–.24	.496
Faculty (1 = FacInt, 2 = FacTrad)	**−.24**	−.46- -.02	.034	**−.23**	−.45- -.01	.040	**−.24**	−.46- -.02	.044	**−.30**	−.53- -07	.012	−.15	−.34–.05	.144	−06	−.23–.11	.488
2nd stage	−.01	−.25–.22	.923	−.05	−.32–.22	.710	−.05	−.32–.22	.703	−.11	−.40–.18	.440	−.11	−.33–.12	.344	−.03	−.22–.16	.746
3rd stage	.04	−.20–.27	.755	.01	−.26–.28	.927	.04	−.24–.31	.926	−.01	−.29–.28	.970	−.07	−.29–.16	.561	−.07	−.26–.12	.446
Personal																		
Age at study start	−.11	−.33–.11	.342				−.12	−.34–.10	.310									
Education mother	.07	−.22–.36	.642				−.01	−.33–.30	.822									
Education father	.23	−.06–.52	.123				.16	−.15–.47	.201									
Living alone	−.08	−.36–.19	.549				−.05	−.36–.26	.814									
Living with friends	.13	−.09–.35	.245				.05	−.20–.31	.600									
Study characteristics																		
Satisfied with supervision	**.41**	.28–.54	.000							**.37**	.24–.51	.000	−.05	−.16–.06	.369			
Failing medical school	**−.84**	−1.22- -.46	.000							**−.96**	−1.33- -.59	.000	.03	−.27–.32	.870			
Study environment																		
Perceived Medical School Stress																		
Medical school is cold and threatening	**−.68**	−.78- -.57	.000										**−.50**	−.60- -.40	.000	**−.38**	−.47- -.29	.000
Worries about work and competence	**−.82**	−.92–.72	.000										**−.60**	−.71- -.50	.000	**−.40**	−.49- -.31	.000
Worries about finance and accommodation	**−.40**	−.50- -.29	.000										**−.31**	−.40- − .22	.000	**-.22**	−.30- -.14	.000
Social support																		
Parents	**.37**	.26–.48	.000										.05	−.05–.15	.336			
Medical School Friends	**.68**	.58–.79	.000										**.30**	.19–.41	.000	**.22**	.12–.31	.000
Other friends	**.45**	.34–.56	.000										.00	−.11–.11	.971			
Other family members	**.32**	.23–.41	.000										.08	−.01–.17	.067	**.08**	.01–.07	.020
Partner	**.06**	.01–.10	.024										**.08**	.05–.12	.000	**.04**	.00–.07	.041
Study administration	**.17**	.08–.25	.000										−.00	−.07–.07	.969			
Perceived exam stress	**−.89**	−.1.00- -.79	.000										**−.38**	−.50- − .26	.000	**-.26**	−.36- -.15	.000
Self-Esteem	**1.71**	1.58–1.85	.000													**.98**	.84–1.12	.000
(Constant)				**7.65**	7.08–8.22	.000	**7.55**	6.56–8.54	,000	**6.73**	5.96–7.49	.000	**6.41**	5.56–7.27	.000	**3.42**	2.64–4.19	.000
R^2^						**.012**			**.011**			**.064**			**.455**			**.533**

**bold** = *p* value less than 0.05

95%*CI* Confidence Interval

Regarding changes in factors associated with SWB, the analyses showed that female students tend to score higher than their male peers on PMSS, both in NORDOC and STUDMED. There was a higher PMSS score in STUDMED for 2nd stage women (mean = 2.87, SD = .72) than NORDOC, (mean = 2.68, SD = .59, *p* = .027), and 3rd stage men (NORDOC mean = 2.35, SD = .60; STUDMED mean = 2.62, SD = .72, *p* = .016). As we lacked PMSS data for NORDOC T1, this study does not allow comparisons to be made between early stage students. For self-esteem, we lacked data from NORDOC T2. We found no difference in self-esteem between NORDOC and STUDMED, but men scored higher (mean = 3.15, SD = .51) than women in NORDOC (T1mean = 2.91, SD = .62, *p* = .007) and in STUDMED’s 1st (men 3.02, SD = .56, women 2.83, SD = .63, *p* = .013) and 3rd stages (men 3.08, SD = .66, women 2.89, SD = .66, *p* = .017).

## Discussion

The present study shows that although the majority of medical students score relatively highly on subjective well-being, subjective well-being was reported to be lower among the medical students of today than two decades ago in all study stages, in particular among the female students.

The effect size in this decrease in well-being among female students today is large at the entry to medical school and small to medium at mid-curriculum and the end of medical school. This indicates that medical school today recruits different female students than they did 20 years ago, whereas there are smaller differences in the samples of male students. The results show that the most important contributors to subjective well-being among the today’s students were self-esteem and social support from medical school friends, a partner and other family members. The most important negative contributors to subjective well-being were perceiving medical school as cold and threatening, worries about work and competence and worries about finances and accommodation, as well as high scores on perceived exam stress.

The decrease in subjective well-being among medical students in general, and among female students in particular, can reflect a general tendency of decreased subjective well-being among adolescents in the general population [49] and the increase in symptoms of anxiety and depression among young women in Norway since 2010 [35, 36]. That the decrease was apparent at baseline/study start for both genders in the current study supports this notion. Accordingly, our results could imply that our sample of medical students is representative of adolescents in general and that the current sample of female medical students represents female adolescents in general.

We found that students at FacInt scored higher on SWB than students at FacTrad and that men scored higher than women. However, the effects of gender and differences between faculties diminished when we controlled for all three factors of perceived medical school stress and social support. This indicates that specific factors related to the study environment and content are important for subjective well-being regardless of national trends in this phenomenon. The findings of the current study are in keeping with earlier findings on the relationship between high PMSS and life satisfaction, a part of subjective well-being [4, 6, 29]. This study emphasizes the independent role of perceived exam stress in addition to PMSS, which captures more than just exam related stress. The current results indicate that other aspects, such as living situation, economy and concerns about one’s ability to thrive, are equally important for medical students’ well-being. This is concordant with earlier findings that students who experienced medical school as interfering less with personal and social life were those who reported high life satisfaction during medical school [6]. There is not only a change in SWB over the last 20 years, there has also been an increase in perceived medical school stress for 2nd stage women and 3rd stage men. This may imply that female students of today are more prone to stress and pressures in medical school than they were 20 years ago. In contrast to the women in 2nd stage who had decreased in subjective well-being, there was no decrease in SWB among 3rd stage men. This can indicate that medical school stress is less influential on subjective well-being among male students in the last phase of their medical studies. Since today’s female students report higher levels of medical school stress in the middle of the curriculum, as well as a decrease in SWB, it seems that medical school today takes a higher toll on female students’ well-being than it did two decades ago. Whether this is due to selection of more vulnerable female students and/or to the fact that medical school is harder for women today remains to be studied. This finding is important in a Scandinavian country, where there are relatively equal gender roles. Given the increasing amount of female students in medical schools world-wide, this is an issue of considerable concern.

Our findings support the significance of a supportive network in student satisfaction and well-being [5, 6, 26], and in particular, our findings show that social support from peers at medical school is most important for subjective well-being among the students, which is in keeping with previous findings on the significance of having a social life outside of studying medicine [6]. Thoits [50] emphasized that the most effective support comes from those who experience the same situations and the same stressors as oneself. Annual reports from the four universities in Norway show that medical students spend more time on curricula activities at the university compared with other students [51, 52]. Undoubtedly, being a period of 6 years, medical study is a significant part of adult life. Cultivating a positive relationship with student peers is thus beneficial for subjective well-being among medical students, as colleague support is important to working doctors [26]. However, a person’s personal network of support constitutes people one feels particularly close to, often rooted in long-standing relationships, such as a partner, family member or close friend [53]. The current study shows that support from a partner, family and friends was important for the current sample of medical students. This has also been shown in previous studies on similar populations [26, 54, 55].

In addition, this study confirms that self-esteem is relevant for life satisfaction among students [29, 30]. The strong association between subjective well-being, self-esteem and the gender difference indicate that self-esteem is more important for subjective well-being among female students. According to an international review, men tend to score higher than females in self-esteem [56], and gender difference in self-esteem has increased in Norway over the last 20 years [56]. Findings on the relationship between self-esteem and performance are somewhat contradictory. One study found that self-esteem was important for academic achievement motivation and in predicting learning performance [57]. In contrast, prospective findings in another NORDOC study showed that the effect of vulnerability (or low self-esteem) on future perceived mastery of clinical work was mediated by preceding skills in medical school [58].

### Strengths and limitations

A major strength is that the study includes validated instruments to ensure high reliability of the reported data and the comparison with representative samples of medical students about 20 years ago. The STUDMED sample is larger (*N* = 1044) compared to the NORDOC cohort, while the latter had higher response rates.

Though the sample was representative based on the study stage, there was a high representation of female participants that could lead to some selection bias; it is therefore important to read the results with these gender differences in mind. As there are only four medical faculties in Norway, the findings may very well be generalised to other Norwegian medical students, since the selection procedures and curricula are fairly similar at the two medical schools not included in the study. Although the study focuses on Norwegian medical students, we believe that the findings could be relevant for faculties and student samples that share the same curricula characteristics, stressors and resources.

Large and relatively representative samples in both samples strengthen our results. As the study is based on cross-sectional data, one must be cautious in making inferences about causality in the associations. Also, the data are based on self-reports, and there have been concerns that self-reported scores on SWB can be influenced by a number of factors, such as the current affective state of the respondent or the order in which the items are presented [59]. However, self-reported well-being measures have shown convergence with non-self-report methods in some senses [60], and we have controlled for the tendency to report high levels of perceived stress due to negative affectivity by controlling for self-esteem (which closely resembles the neuroticism trait). The inclusion of self-esteem in the regression in Model 5 does not, to a large degree, change the variables that come out as being associated with SWB, which indicates that the variables are significant from individuals with both high and low self-esteem.

Though the study model explains a fair amount of the variance in SWB, there are other factors that might be influential, such as having a doctor parent [61], avocational and extracurricular activities [5, 6], physical training [5, 62] and religion [63]. Unfortunately, the current study did not include these variables.

It is important to note that the students at FacTrad were having an exam period at the time of the data collection. Though the sample was representative based on the study stage, albeit not gender, the exam session itself could have caused a self-selection of respondents based on gender or faculty. This could have affected the response rate on the survey and the participants’ SWB scores. Though students at FacTrad are used to frequent exams, the frequency of exams at FacTrad made it challenging to collect data beyond either the preparation of or during an exam period. Accordingly, the period of data collection was based on recommendations from the faculty administration and student union at both faculties. Preliminary analyses showed no difference between the faculties on perceived exam stress included in the study, nor were the interactions between faculties, perceived exam stress and other significant variables associated with SWB. This could indicate that either factors other than the current exam or the identified variables were relevant to explain the differences between FacInt and FacTrad on subjective well-being.

## Conclusions

Though there has been a decrease among medical students over the last two decades, Norwegian medical students still score relatively high on subjective well-being, which indicates that they thrive at their respective faculties. As the lower levels of subjective well-being were most evident at the stage of entering medical school, this can reflect a decrease in the overall well-being of today’s youth in general, in particular, among young women. The study underlines the impact of individual differences, as well as factors in the study environment, medical school and medical curricula on students’ subjective well-being. It appears that targeting factors related to students’ support system, characteristics of the student environment, economy and accommodation, as well as perceptions of work and personal competence, is particularly important. Accordingly, it is vital that both study demands and positive influencers and resources are included in future empirical research investigating medical students’ health and well-being. As for medical schools, they need to go beyond the medical curriculum to teach graduates the skills necessary to develop strategies to promote their own well-being to effectively stimulate learning, competence and personal as well as professional growth.

## Abbreviations

BCI: Basic Character Inventory
ColdThreat: Medical school is cold and threatening
FacInt: Faculty with integrated curriculum
FacTrad: Faculty with traditional curriculum
FinAcc: Worries about finance and accommodation
PBL: Problem based learning
PMSS: Perceived Medical School Stress
SSadm: Social support from the study administration
SSfam: Social support from other family members
SSmed: Social support from medical school friends
SSofr: Social support from other friends
SSpar: Social support from parents
SSptn: Social support from partner
SWB: Subjective well-being
WorkComp: Worries about work and competence
